# Unprovoked Isolated Pulmonary Embolism and Graves’ Disease in a Patient With Dyspnea: A Case Report

**DOI:** 10.7759/cureus.24972

**Published:** 2022-05-13

**Authors:** Roshan Bisural, Deepak Acharya, Samaj Adhikari, Baikuntha Chaulagai, Arjun Mainali, Tutul Chowdhury, Nicole Gousy

**Affiliations:** 1 Internal Medicine, Interfaith Medical Center, Brooklyn, USA; 2 Medicine, American University of Antigua, New York City, USA

**Keywords:** hyperthyroid, severe dyspnea, unprovoked pulmonary embolism, acute pulmonary embolism, graves´disease

## Abstract

Graves’ disease is a commonly diagnosed disease with a plethora of manifestations that can lead to its diagnosis. One of the rarer presentations of Graves’ disease is hypercoagulability with the development of spontaneous venous thrombosis. In patients presenting with unprovoked pulmonary embolism, we suggest evaluating the patient’s thyroid function tests as a potential underlying cause. To bring this issue to attention, we are presenting a rare case of isolated spontaneous pulmonary embolism development secondarily to underlying Graves’ disease.

## Introduction

Coagulopathy is one of the rare manifestations of hyperthyroidism; however, there have been multiple isolated reports of cerebral venous sinus thrombosis associated with a hyperthyroid state [1,2]. Incidentally, there are even fewer case reports reported on the development of pulmonary embolisms (PEs) associated with Graves' disease [3-5]. Hypercoagulability can be observed in hyperthyroid states and is attributed to elevated levels of serum fibrinogen, von Willebrand factor (vWF), and factor VII in addition to compounding endothelial dysfunction and decreased fibrinolytic activity [6-8]. Diagnostic workup of Graves’ disease is frequently neglected in patients presenting with unprovoked PE. To bring awareness to this important differential diagnosis, we present a case of unprovoked PE in a patient with Graves’ disease.

## Case presentation

A 58-year-old female with a past medical history of hypertension and bronchial asthma presented to the emergency department with shortness of breath for one week. Her shortness was getting progressively worse and further aggravated with exertion. She attempted to medicate herself using an albuterol inhaler which provided no relief to the symptoms. She denied any recent history of immobilization, travel, past medical history of malignancy or recent surgery. She had no significant family history, including a family history of blood clot formation or clotting disorders. She currently smokes a half pack of cigarettes daily for over 20 years but denied alcohol consumption or the use of recreational drugs.

At triage, her vitals signs were as follows: pulse rate 133 beats per minute, blood pressure 163/78 mmHg, respiratory rate was 18 per minute, body temperature of 98.6 F and oxygen saturation of 83% on room air and she maintained oxygen saturation of 97% on two liters of nasal cannula. Physical examination was negative for pallor, icterus, lymphadenopathy, clubbing, cyanosis, edema or dehydration. Chest examination revealed equal vesicular breath sounds bilaterally with no added sounds. A cardiovascular exam revealed a normal-sounding S1 and S2 with sinus tachycardia and a normal rhythm. There were no murmurs, rubs or gallops appreciated. Laboratory evaluation showed a normal WBC count 7.1k/µL, an elevated brain natriuretic peptide (BNP) (800.60 pg/mL) and a normal D-dimer (164 ng/mL). Significant lab findings are listed in Table 1 with elevated thyroid-stimulating immunoglobulin (TSI ) confirming the diagnosis of Graves' disease.

**Table 1:** Patient’s thyroid profile with immunology taken during patient admission TSH: Thyroid-stimulating hormone, µLU/mL: micro-international units per milliliter, ng/dL: nanograms per deciliter, IU/mL: international unit per milliliter

Test	Ref Range and Units	Values
TSH	0.450-4.500 µLU/mL	<0.005
Thyroxine (T4) Free, Direct	0.82-1.77 ng/dL	3.22
Triiodothyronine (T3)	71-180 ng/dL	303
Thyroid peroxidase antibodies	0-34 IU/mL	68
Thyroid stimulating immunoglobulin	0.00-0.55 IU/mL	2.51

**Figure 1:**
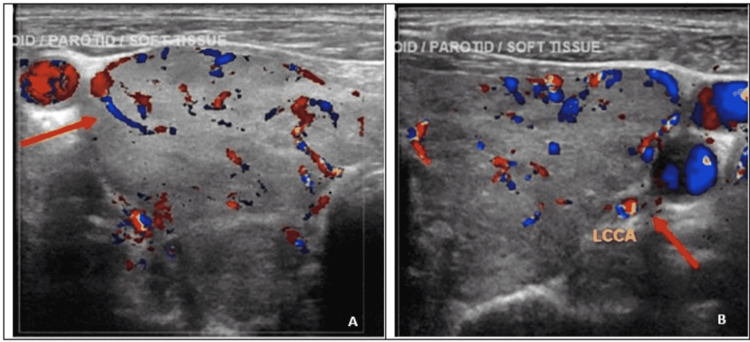
Right (A) and left (B) thyroid gland showing thyroidmegaly with heterogenous echotexture and increased vascularity (red arrows)

Chest x-ray plain film revealed chronic appearing interstitial lung changes. Electrocardiogram was significant only for sinus tachycardia. Ultrasound of the thyroid showed heterogeneous echotexture and increased vascularity throughout the gland without discrete nodules (Figures 1A, 1B).

**Figure 2:**
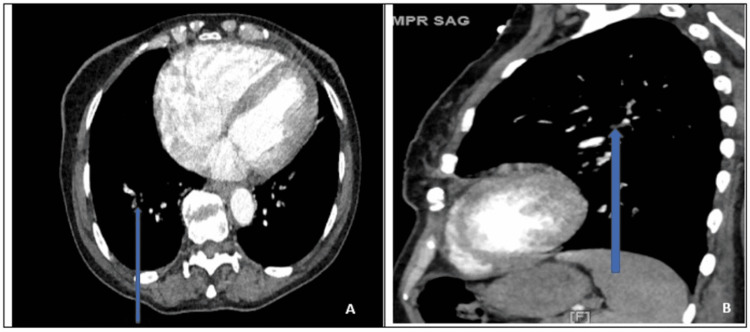
CTPA in axial (A) and sagittal (B) sections with blue arrows pointing towards the filling defect in the subsegmental artery in the right lower lobe (blue arrows)

As per the Burch-Wartofsky Point Scale, her total score was 20, unlikely to represent a thyroid storm. Transthoracic echocardiography revealed Grade I diastolic dysfunction with left ventricular ejection fraction (LVEF) of 60%-65%, a pulmonary systolic arterial pressure of 50-55 mmHg, and a mild to moderate tricuspid regurgitation. PE was highly suspected and a contrast-enhanced CT scan was performed, which showed embolism in the subsegmental artery in the right lower lobe (Figures 2A, 2B).

The patient was started on enoxaparin. A bilateral lower extremity Doppler study did not show any evidence of deep vein thrombosis. Meanwhile, the work up for other causes of hypercoagulability including protein C antigen and free protein S were negative. Immunologic workup including antinuclear antibodies (ANA), c-ANCA, p-ANCA, Ds-DNA, anti-SS-A, anti-SS-B, smith and RNP antibodies were also negative. The patient was continued on enoxaparin 40 mg subcutaneously twice a day which was later switched to an oral anticoagulant. To account for the newly diagnosed Graves' disease, the patient was started on Propranolol 20 mg three times a day and methimazole 10 mg daily. Her shortness of breath and tachycardia improved during the hospital stay and she was discharged on the eighth day of hospital admission with an oral anticoagulant.

## Discussion

Graves’ disease is a commonly diagnosed disease and is seen more commonly in women, with an overall incidence of 2.5% [9]. The incidence of PE in those with Graves’ disease, however, was shown to be 0.08%, according to a longitudinal study done by Lin et al. over a five-year period [9]. They additionally concluded that the risk of having a PE was 2.31 times greater for patients with hyperthyroidism compared to those without a pre-existing diagnosis of hyperthyroidism within the same five-year period (95% confidence interval 1.20-4.45, P=0.012) [9].

Unprovoked deep venous thrombosis (DVT) has been frequently observed among patients with hyperthyroidism in previous studies [4,10]. The pathophysiological mechanism of hypercoagulability among patients with Graves’ disease is associated with the elevation of plasminogen activator inhibitor (PAI-1) and vWF [11,12]. In a case-control study done by Zaane et al., increasing serum level of fT4 was also found to be correlated with an increased risk of venous thrombosis [10]. The derangements of these proteins are thought to create a hypercoagulable state that can endorse the development of venous thrombosis formation in sinus, cerebral or deep vein DVTs [10].

 Previous case reports with PE in patients with Graves’ disease had concomitant deep vein thrombosis in contrast to isolated PE in our case [3,4]. While it is common for DVTs to embolize and leads to PEs, there is minimal literature describing isolated PE development in patients with Graves’ disease [3,4]. However, our case is in agreement with the case reported by Lashari et al., with negative venous ultrasonography of lower extremities in the setting of acute PE without potential inciting factors or provocation [13].

Lee et al. found nearly half of patients with PE had concomitant deep vein thrombosis [14]. Nevertheless, isolated PE in the absence of prolonged immobilization, congestive heart failure, or recent surgery, should be worked up for other underlying causes including hereditary or acquired causes of hypercoagulability [3,14,15]. Notably, the thyroid state should also be evaluated in this list. Routine use of oral anticoagulants is, however, not recommended in patients with hyperthyroidism given the low prevalence of thrombotic events [15].

## Conclusions

Graves’ disease can be complicated by thromboembolic events, regardless of clinical or subclinical hyperthyroidism. Our study suggests a thyroid workup should be done in addition to conventional thrombophilia workup, in the assessment of the patients with unprovoked isolated PE. Since this test is so readily available, it is not unreasonable to consider evaluating the thyroid state as a possible explanation for an unprovoked venous thrombosis formation.
